# Supplementary material for: Growth and Decay of Fecal Indicator Bacteria and Changes in the Coliform Composition on the Top Surface Sand of Coastal Beaches during the Rainy Season
Source: Microorganisms. 2023 Apr 20;11(4):1074. doi: 10.3390/microorganisms11041074 (PMC10145847; doi:10.3390/microorganisms11041074)
Supplement: Supplementary file 1 [file microorganisms-11-01074-s001.zip › microorganisms-2329277-supplementary.pdf]

Supplementary Figure and Table

Growth and decay of fecal indicator bacteria and changes in the coliform composition on the top surface sand of coastal beaches during the rainy season

Soichiro Tamai, Hiroshi Shimamoto, Kei Nukazawa, Yoshihiro Suzuki*

Department of Civil and Environmental Engineering, Faculty of Engineering, University of Miyazaki,

Miyazaki 889-2192 , Japan; hh17057@student.miyazaki-u.ac.jp (S.T.); shimamo-to@cc.miyazaki-u.ac.jp (H.S.);

nukazawa.kei.b3@cc.miyazaki-u.ac.jp (K.N.)

*Correspondence: ysuzuki@cc.miyazaki-u.ac.jp; Tel.: +81-985-58-7339

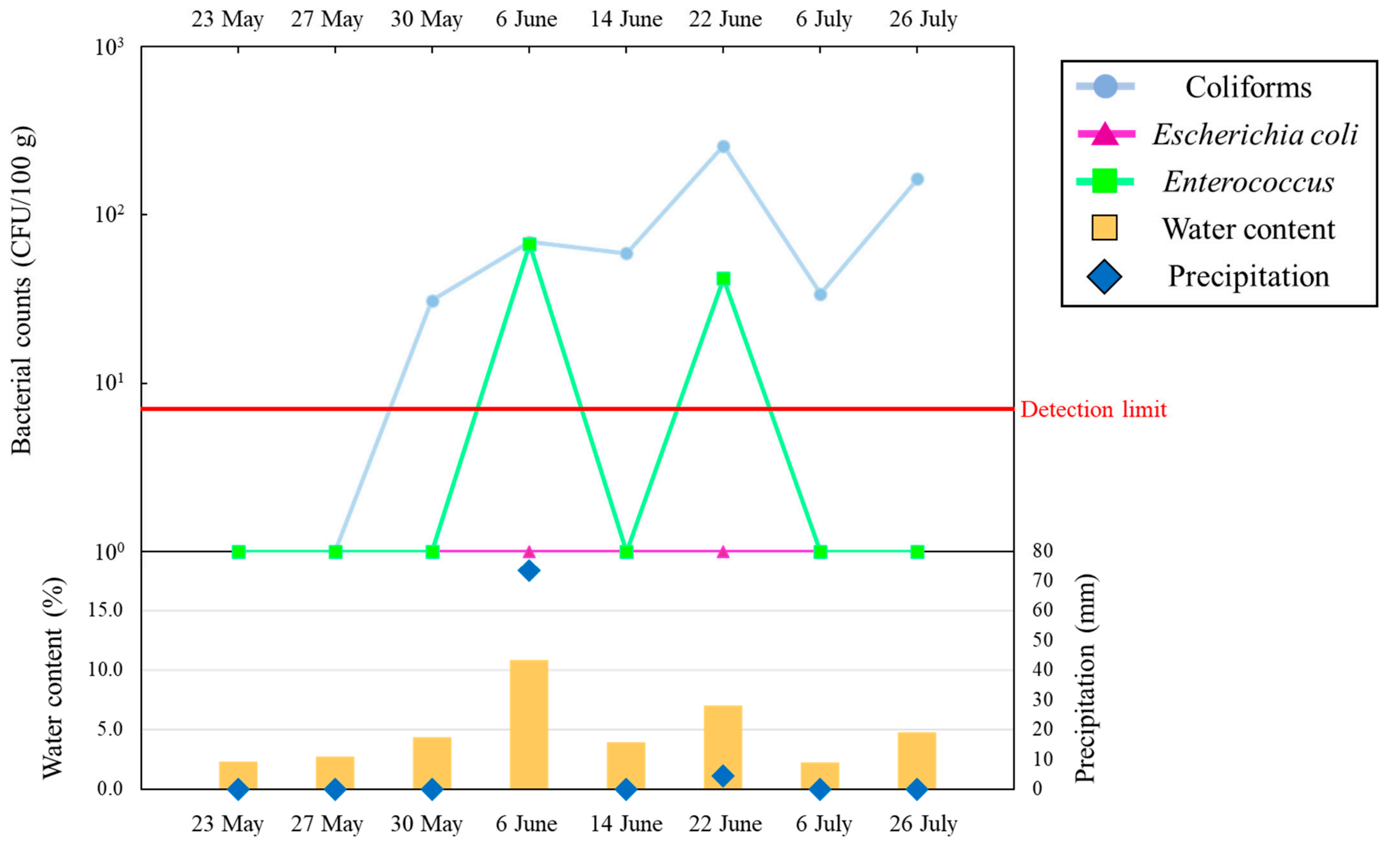

Figure S1. Variation of *Escherichia coli*, coliform, and *Enterococcus* counts in supratidal 10 cm sand; water content; and precipitation at Kizaki Beach.

Supplementary Figure and Table

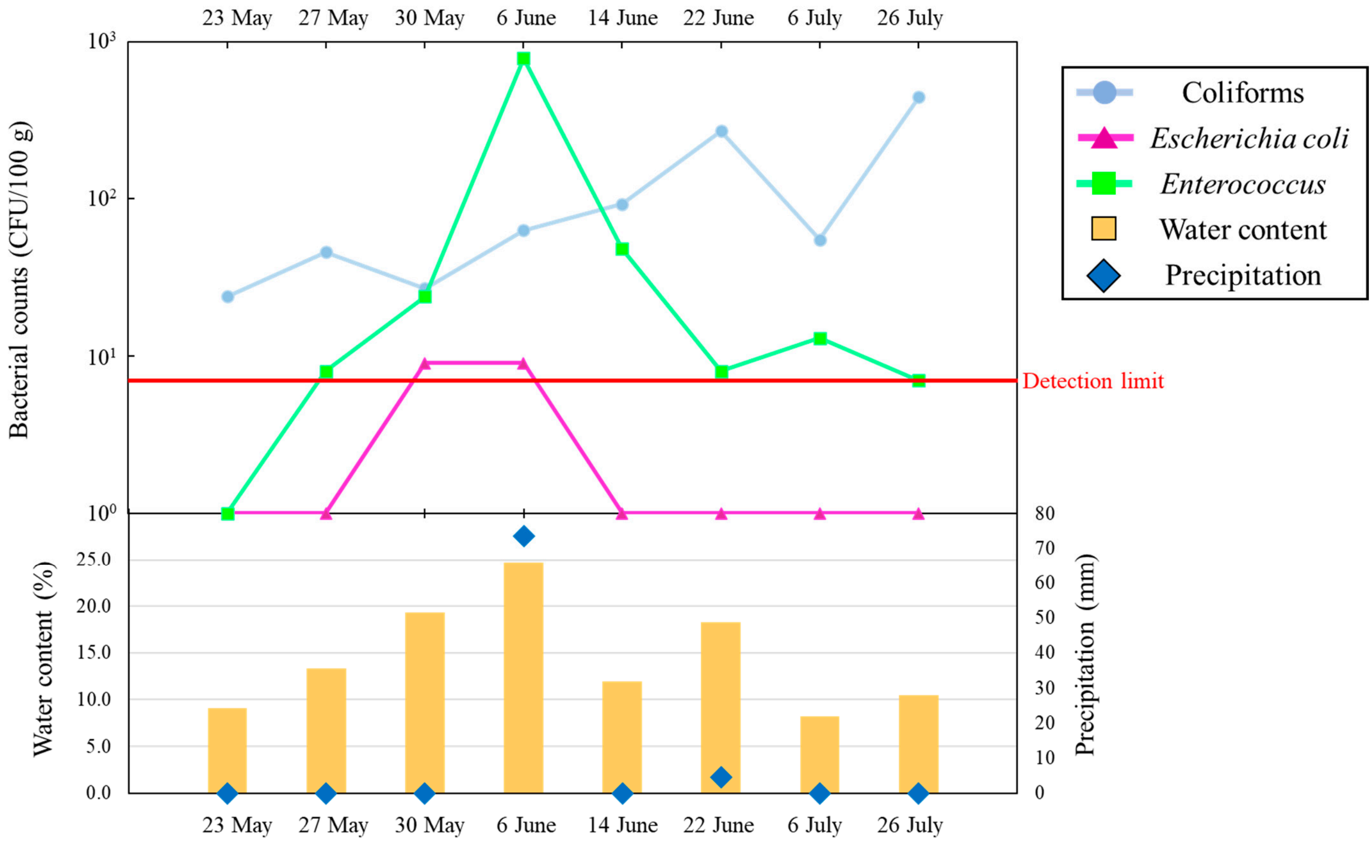

Figure S2. Variation of *Escherichia coli*, coliform, and *Enterococcus* counts in supratidal 10 cm sand; water content; and precipitation at Shirahama Beach.

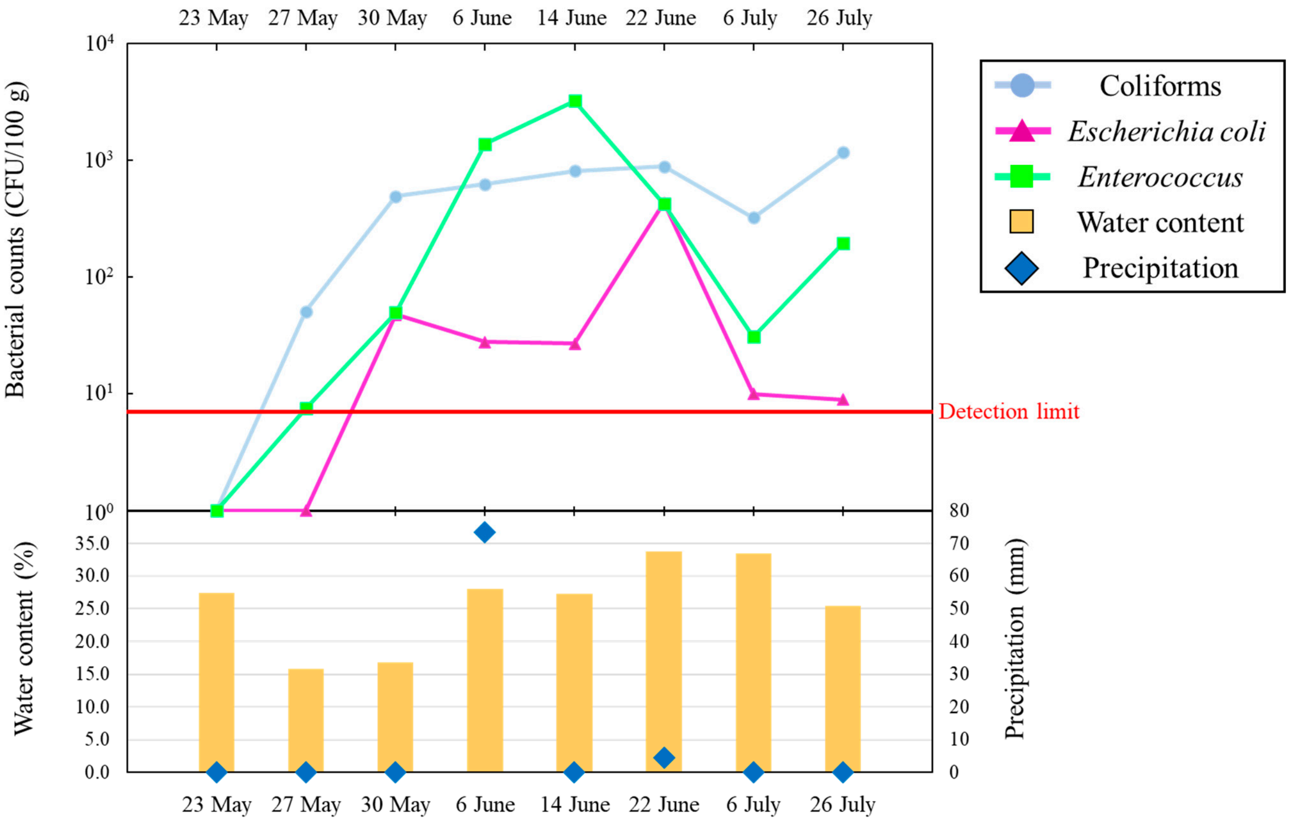

Figure S3. Variation of *Escherichia coli*, coliform, and *Enterococcus* counts in intertidal 10 cm sand; water content; and precipitation at Kizaki Beach.

Supplementary Figure and Table

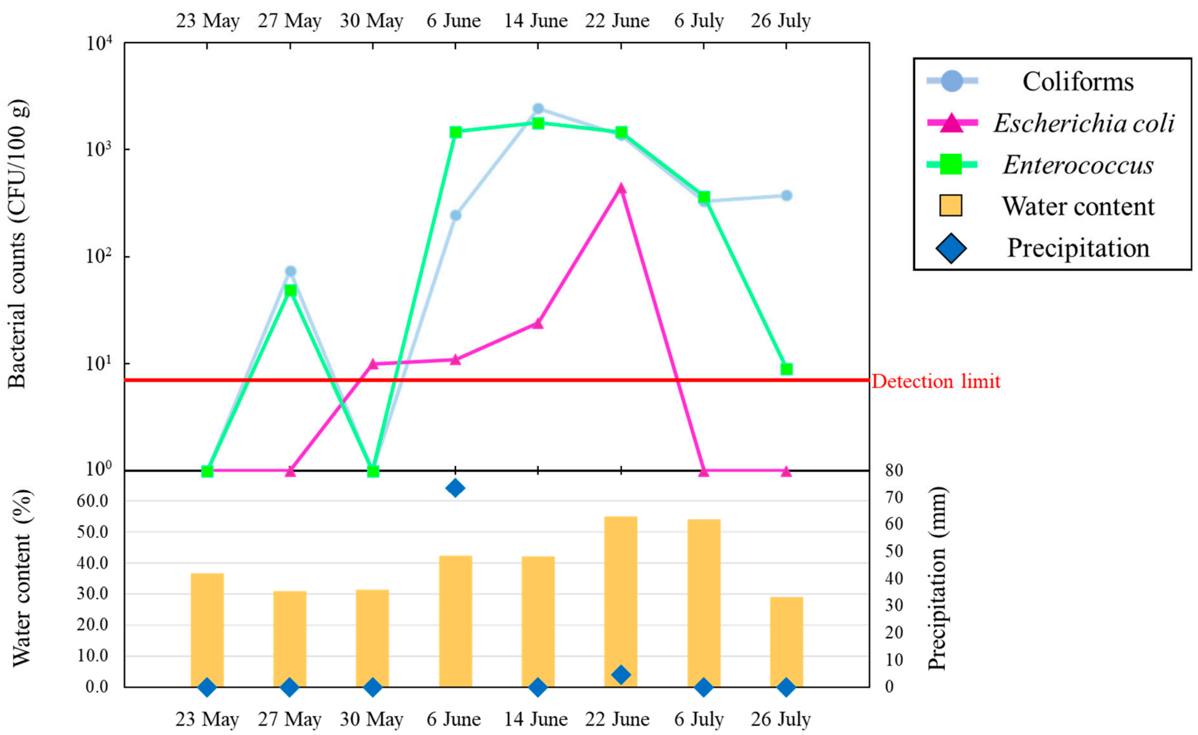

Figure S4. Variation of *Escherichia coli*, coliform, and *Enterococcus* counts in intertidal 10 cm sand; water content; and precipitation at Shirahama Beach.

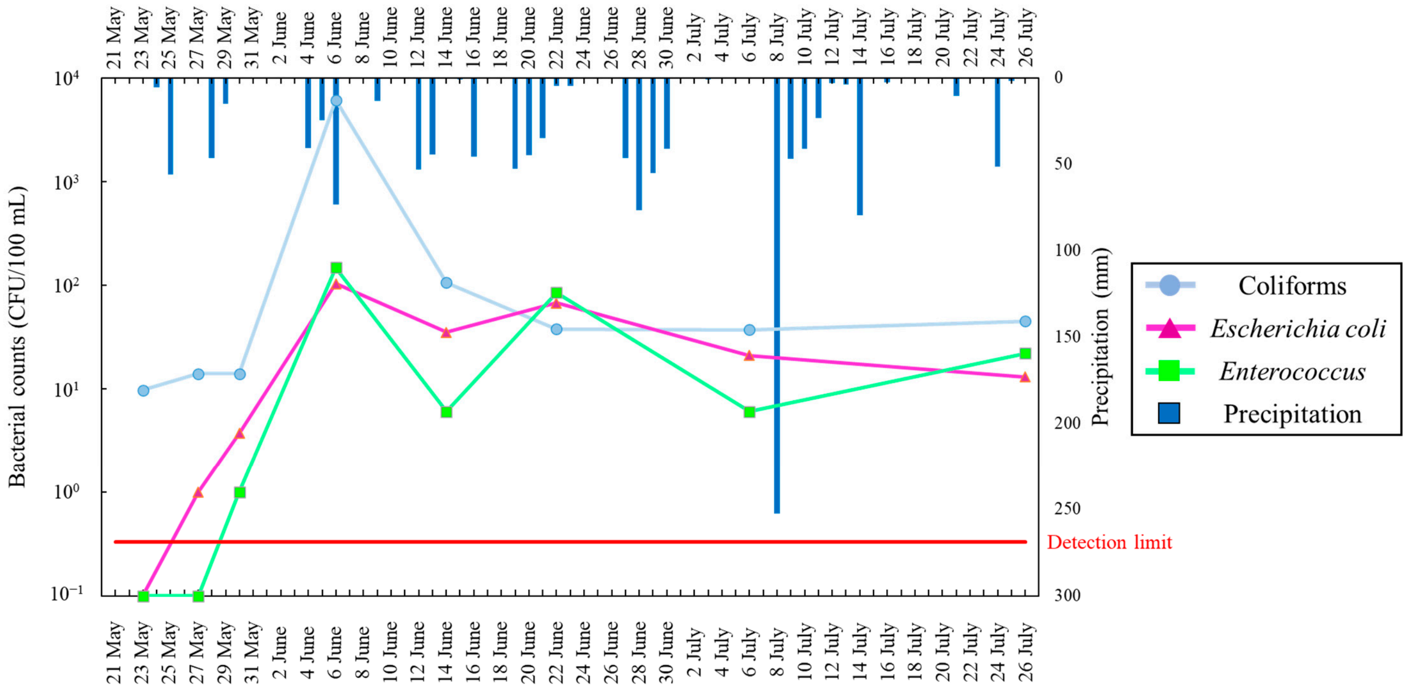

Figure S5. Variation of *Escherichia coli*, coliform, and *Enterococcus* counts in the seawater; precipitation at Kizaki Beach.

Supplementary Figure and Table

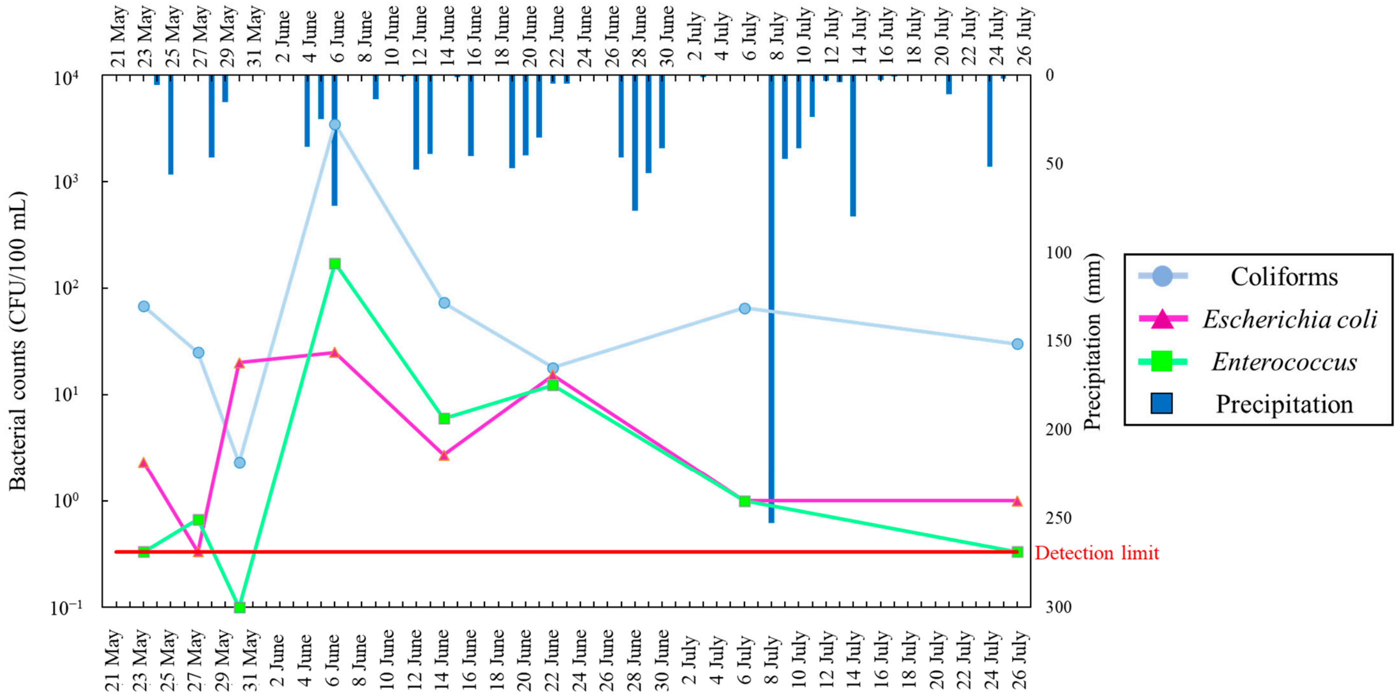

Figure S6. Variation of *Escherichia coli*, coliform, and *Enterococcus* counts in the seawater; precipitation at Shirahama Beach.

Supplementary Figure and Table

Table S1. Sunshine duration, wind speed, and precipitation at a meteorological station (31°48'10.8"N 131°27'36.0"E) near the sampling site.

	Sunshine duration (h)	Average wind speed (m/s)	Maximum wind speed (m/s)	Maximum instantaneous wind speed (m/s)	Precipitation (mm)	Average temperature (°C)	Maximum temperature (°C)	Minimum temperature (°C)
23-May	8.8	1.7	3.8	6.2	0	21.7	26.1	18.5
27-May	2.9	1.3	3.3	6.7	0	24.4	28.1	21.5
30-May	9.6	1.8	3.9	7.2	0	23.8	27.4	20.4
31-May	11.9	1.6	4.3	8.2	0	24	28.2	19.2
1-Jun	0	1.3	2.6	5	0	21.4	23.3	18.5
2-Jun	11.9	2.1	4.3	8.8	0	20.9	24.8	17
6-Jun	0	2.6	5.4	11.2	73.5	20.5	21.2	19.3
7-Jun	5.5	1.7	4.5	7.3	0	23.3	27.4	20.8
8-Jun	4.4	1.4	3	4.5	0	23.9	26.8	21.7
9-Jun	4.6	1.3	3.3	5.3	13.5	23	26.2	20.9
10-Jun	9.3	1.6	4.4	6.8	0	23.6	27	20.3
11-Jun	1.6	1.4	3.4	5.7	0.5	24.6	28.1	21.9
12-Jun	0	1.2	3.8	8	53	23.6	25.1	22.6
13-Jun	0	0.9	2.9	4.4	44.5	23	23.9	21.1
14-Jun	10.2	1.4	3.7	7.3	0	24.2	28.9	20.2
15-Jun	5.4	1.5	3.7	7.9	1	24.6	28.8	21
16-Jun	0	1.7	5.6	11	45.5	24.6	28.1	23
17-Jun	0	1.3	4.6	11.9	0	23.1	26.3	20.5
20-Jun	0	1.4	4.3	9.5	45	24.5	28.5	23
21-Jun	3.3	1.3	4.3	8.5	35	26.8	32	24.3
22-Jun	1.7	1.2	3.6	8.1	4.5	26.5	31.5	23.7
23-Jun	9.9	2.3	5.1	11.7	4.5	29.2	33.9	25.4
24-Jun	3.6	1.9	5.1	10.5	0	29.5	32.9	24.7
25-Jun	2.1	2.8	6.1	13.6	0	25.8	30.5	20.1
26-Jun	12.7	2.5	5.7	11	0	25	31.4	19.6
27-Jun	1	1.1	2.8	4.9	46.5	19.5	22.7	18.2
28-Jun	0	1.2	3.7	7.1	76.5	24.4	27.3	19.4
29-Jun	0.1	1.1	3.8	8.3	55	24.6	27.3	22.8
30-Jun	0.9	0.9	3.3	6.3	41	23.2	26.5	21.1
1-Jul	4.8	1.3	3.4	7	0	25.5	31.2	20.9
2-Jul	9.4	1.4	3.1	6.8	0	28.3	33.7	22.8
3-Jul	10.1	1.4	3.5	7.7	1	29.4	34.9	25.4
4-Jul	7.7	1.2	3.4	6.3	0	28.1	33.1	24
5-Jul	12.3	1.6	4.1	8.8	0	28	32.6	23.9
6-Jul	13.7	1.7	4	7.1	0	27	31.3	23.3
10-Jul	0.8	0.8	3	7.1	41	25.4	27.9	24.3

12-Jul	8.8	1.4	3.8	10.6	3	28.7	34.8	25.2
14-Jul	2.7	1.5	4.4	10.3	79.5	27.1	31.7	24.3
15-Jul	10.4	1.8	4.5	8.3	0	26.1	29.6	21.4
26-Jul	10.7	1.6	4.3	7.5	0	26.7	30.2	23.8

Table S2. Bacterial counts, water content and surface temperature in supratidal top surface sand; precipitation at Kizaki Beach.

	Total Coliform (CFU/100g)	*Escherichia coli* (CFU/100g)	*Enterococcus* (CFU/100g)	Water content (%)	Temperature (°C)
23-May	439	0	0	0.5	47.6
27-May	0	0	0	0.4	27.8
30-May	15	0	0	0.6	36
31-May	56	0	0	0.5	45.3
1-Jun	453	0	121	2.7	23.9
2-Jun	35	0	20	0.2	40.5
6-Jun	749	24	330	13.2	22
7-Jun	1629	65	109	5.6	30.2
8-Jun	90	0	0	0.1	35.2
9-Jun	47	7	117	1.1	39.4
10-Jun	20	0	14	0.5	45.8
11-Jun	62	0	0	0.2	41
12-Jun	1010	0	740	13.3	26.2
13-Jun	3524	8	91	9.7	26.6
14-Jun	1243	0	0	0.3	43.5
15-Jun	26	0	0	0.4	42.1
16-Jun	1486	147	116	11.6	24.1
17-Jun	2226	0	92	4.0	24.3
20-Jun	686	0	129	10.1	25.1
21-Jun	4420	289	171	6.0	30.6
22-Jun	1955	8	65	5.0	28.5
23-Jun	79	0	0	0.3	44.1
24-Jun	8	0	0	0.2	41.5
25-Jun	21	0	7	0.2	36.1
26-Jun	39	0	7	0.1	51
27-Jun	94	0	7	12.4	23.2
28-Jun	927	8	306	8.5	27.6
29-Jun	3732	15	103	7.0	31.3
30-Jun	3983	0	394	9.5	24.8
1-Jul	1913	0	0	0.4	41

2-Jul	23	0	0	0.2	41.5
3-Jul	643	0	20	0.4	39.6
4-Jul	21	0	7	0.4	54
5-Jul	7	0	0	0.1	51.5
6-Jul	34	0	0	0.1	50.5
10-Jul	2342	53	13	6.7	36.4
12-Jul	76	0	0	0.9	42.5
14-Jul	4698	0	1064	2.9	29.4
15-Jul	35	0	0	0.2	52
26-Jul	21	0	0	0.1	47.4

Supplementary Figure and Table

Table S3. Bacterial counts, water content and surface temperature in supratidal top surface sand; precipitation at Shirahama Beach.

	Total Coliform (cfu/100g)	*Escherichia coli* (cfu/100g)	*Enterococcus* (cfu/100g)	Water content (%)	Temperature (°C)
23-May	1050	0	0	0.7	47.5
27-May	4279	0	0	9.8	24.2
30-May	246	480	49	18.2	32.5
31-May	643	0	0	8.4	32.5
1-Jun	312	0	124	3.6	23.3
2-Jun	28	0	0	0.6	35.8
6-Jun	189	8	991	20.1	22
7-Jun	435	151	1300	12.6	30.1
8-Jun	121	0	7	2.3	32.5
9-Jun	699	1536	2844	11.9	33.2
10-Jun	25	246	590	2.1	37.6
11-Jun	57	0	7	1.1	35
12-Jun	818	364	566	13.6	27.2
13-Jun	3205	450	132	14.5	25.5
14-Jun	715	8	44	8.6	35
15-Jun	115	0	0	0.7	41.5
16-Jun	325	914	515	17.5	25.4
17-Jun	4911	31	74	11.6	25.1
20-Jun	4933	26	15	15.3	24.7
21-Jun	1948	199	141	11.5	32
22-Jun	1123	16	1882	12.4	29.2
23-Jun	2118	9	15	11.8	33.1
24-Jun	107	0	0	1.4	37.7
25-Jun	251	0	0	0.5	37.6
26-Jun	83	0	0	0.6	43.4
27-Jun	415	0	547	15.5	22.1
28-Jun	5521	0	15	11.3	25.8
29-Jun	852	0	121	10	29.8
30-Jun	3194	33	77	14	24.7
1-Jul	1570	8	65	8.6	32.7
2-Jul	536	0	0	0.9	36.3
3-Jul	7280	375	3075	7.1	34.4
4-Jul	86	0	19	0.4	43.1
5-Jul	20	0	0	0.3	46
6-Jul	55	0	13	0.3	33.1
10-Jul	785	19	359	20	34

12-Jul	3596	12	780	6.4	36.2
14-Jul	3448	0	64	9	31.7
15-Jul	541	0	0	0.7	42.2
26-Jul	351	0	1492	6.2	37.5

Supplementary Figure and Table

Table S4. Composition of coliforms in supratidal top surface sand at Kizaki Beach.

	31-May	1-Jun	2-Jun	7-Jun	8-Jun	9-Jun	10-Jun	11-Jun	12-Jun	13-Jun	15-Jun	16-Jun	17-Jun	20-Jun	21-Jun	23-Jun	24-Jun	25-Jun	26-Jun	27-Jun	28-Jun	29-Jun	30-Jun	1-Jul	2-Jul	3-Jul	4-Jul	5-Jul	10-Jul	12-Jul	14-Jul	15-Jul
Acinetobacter															1	1			1		1											
Aeromonas									1																							
Cedecea																					1								1			
Citrobacter	1								3	1	1			18									20			1			3		1	
Cronobacter				1				2				1		1							1		2						2			
Cupriavidus																														2		
Delftia																							1									
Enterobacter	10			10					5	19		5	15	2	13	2		1	3	3	18	29	3	30	3	26			16	10	26	3
Escherichia				3																1												
Klebsiella													9		2														1			
Kluyvera				1					2														1									
Kosakonia				7																	2											
Leclercia	2			2	12		2	7	8	6	1	20	4	2		1	1	2		2	2	1	1				1	1	2		1	3
Lelliottia	1	27													13	2										1						
Mycoplasma																														1		
Ochrobactrum															1											1						
Paenibacillus																			1											2		
Pantoea	4	2	5		1				5	2	1	1	1							8	3					1			1	7		
Providencia				1										4																		
Pseudomonas									1			1				1															1	
Raoultella													1																1			
Serratia				5					4																				3			

Supplementary Figure and Table

Table S5. Composition of coliforms in supratidal top surface sand at Shirahama Beach.

	31-May	1-Jun	2-Jun	7-Jun	8-Jun	9-Jun	10-Jun	11-Jun	12-Jun	13-Jun	15-Jun	16-Jun	17-Jun	20-Jun	21-Jun	23-Jun	24-Jun	25-Jun	26-Jun	27-Jun	28-Jun	29-Jun	30-Jun	1-Jul	2-Jul	3-Jul	4-Jul	5-Jul	10-Jul	12-Jul	14-Jul	15-Jul
Aeromonas	1																															
Achromobacter		1									1																					
Acinetobacter												1	2			1		22	1		1			2	1							
Cedecea														2																		
Citrobacter				3					15	3		14		1									1									
Cronobacter																				1	1		11									
Enterobacter	22	12		7	1	2	1	2	5	24		6	28	26	3	12	17	1	1	15	18	28	7	3	7	7	1		29	26	10	7
Erwinia																1																
Escherichia				4		27			1			2			2					1			4			3						
Klebsiella												1								3			1	23		19	1		1			
Kluyvera																9							1									
Kosakonia																				2					1							1
Leclercia	3	2		10	1		2	4	1		10	3			13				1		5	2		1			6	2		1		6
Lelliottia								1			1				7								2	1								
Lysinibacillus																											1					
Mycoplasma							1																									
Ochrobactrum															2																	2
Paenibacillus																			1								1					
Pantoea		9	4		11			3		1	1	3				1		7	6	3					16	1	1	1				11
Pseudomonas	3	1			1						1								1		4						1				1	
Raoultella																							3									
Rhizobium																			1													
Salmonella																				3												
Serratia				2	2	1			1						1															3	19	
Stenotrophomonas														1						1												

Supplementary Figure and Table

Table S6. List of the major pathogenic bacteria of the bacterial genus isolated from the two beaches and the diseases they cause. Only major bacterial genera present at >5.0% of the relative abundance are shown.

Genus	Major pathogenic bacteria (species)	Lists of diseases	Reference
Enterobacter	*Enterobacter cloacae*<br>*Enterobacter hormaechei*<br>*Enterobacter agglomerans*	Urinary tract infections, Respiratory infections, Soft tissue infections, Osteomyelitis, and Endocarditis.	27
Leclercia	*Leclercia adecarboxylata*	Endocarditis, Bacteremia, Cellulitis, Urinary tract infections, Pneumonia, and Bacterial peritonitis.	28
Citrobacter	*Citrobacter freundii*<br>*Citrobacter koseri*<br>*Citrobacter braakii*	Urinary tract infections, Intra-abdominal infections, surgical site infections, skin and soft tissue infections, and respiratory tract infections.	29
Lelliottia	*Lelliottia nimipressuralis*	Skin and soft tissue infection.	30
Pantoea	*Pantoea agglomerans*<br>*Pantoea dispersa*	Neonatal infection, Wound infections, Synovitis, Septic arthritis, Osteomyelitis, Bloodstream infections, Peritonitis, Cholelithiasis, Endophthalmitis, Endocarditis, Dacryocystitis, Urinary tract infection, Meningitis, Brain abscess, and Respiratory tract infections.	31
Klebsiella	*Klebsiella pneumoniae*<br>*Klebsiella ozaenae*<br>*Klebsiella rhinoscleromatis*	Urinary tract infections, Pneumonia, Septicemia, Wound infections, Nosocomial infections in intensive care unit patients, and Neonatal septicemia	32
Escherichia	*Diarrheagenic Escherichia coli*	Traveler's diarrhea, hemorrhagic colitis and hemolytic-uremic syndrome, persistent diarrhea, and watery diarrhea of infants.	33

Table S7. Results of the logistic regression model for the proportion of *Enterobacter*.

	Kisaki Beach		Shira Beach	
	Coefficient	p>|z|	Coefficient	p>|z|
Water content rate	-0.0287	0.837	0.0713	0.438
Temperature	-0.0172	0.792	-0.0515	0.571
Constant	0.1787	0.949	0.6179	0.861
Observations	31		32	
Log likelihood	-16.733		-15.718	
